# Arf1 facilitates mast cell proliferation via the mTORC1 pathway

**DOI:** 10.1038/s41598-022-26925-1

**Published:** 2022-12-24

**Authors:** Yui Kotani, Mami Sumiyoshi, Megumi Sasada, Toshio Watanabe, Satoshi Matsuda

**Affiliations:** 1 Department of Cell Signaling, Institute of Biomedical Science, Kansai Medical University, Hirakata, Osaka 573-1010 Japan; 2 Department of Biological Science, Graduate School of Humanities and Sciences, Nara Women’s University, Nara, 630-8506 Japan

**Keywords:** Biochemistry, Immunology

## Abstract

Mast cells are one of major players in allergic responses. Mast cell activation via the high affinity IgE receptor (FcεRI) causes degranulation and release of de novo synthesized proinflammatory cytokines in a process that involves vesicle trafficking. Considering that the GTPase ADP-ribosylation factor 1 (Arf1) orchestrates and maintains membrane traffic and organelle structure, it seems likely that Arf1 contributes to mast cell activation. Actually, it has been reported that pharmaceutical blockade of the Arf1 pathway suppresses cytokine secretion and mast cell degranulation. However, physiological roles of Arf1 in mast cells remain elusive. Here, by using a genetic approach, we demonstrate that Arf1 is required for optimal mTORC1 activation upon IL-3 and facilitates mast cell proliferation. On the other hand, contrary to our expectation, Arf1-deficiency had little impact on FcεRI-induced degranulation nor cytokine secretion. Our findings reveal an unexpected role of Arf1 in mast cell expansion and its potential as a therapeutic target in the mast cell proliferative disorders.

## Introduction

Mast cells play a critical role in both acute anaphylaxis and chronic allergic inflammation, which are mainly mediated by the high-affinity IgE receptor (FcεRI)^1–3^. Crosslinking of IgE-bound FcεRI by its cognate antigen triggers mast cell activation, leading to the immediate degranulation and release of preformed mediators like histamine from secretory granules as well as de novo synthesis of cytokines and chemokines, which are secreted after vesicular trafficking via the ER and Golgi complex^4^.

ADP-ribosylation factor (Arf) proteins are small GTPases belonging to Ras superfamily, and orchestrate intracellular protein trafficking under the control of their activators, guanine nucleotide exchange factors (GEFs), and inhibitors, GTPase-activating proteins (GAPs)^5,6^. Mice have six Arf isoforms (Arf1-Arf6), which can be divided into three classes based on sequence homology: class I (Arf1, Arf2, and Arf3), class II (Arf4 and Arf5), and class III (Arf6)^6^. Given that the intracellular protein trafficking system controls a diverse array of cellular responses including proliferation, differentiation, and cell migration, one can assume that this is also the case with the immune responses. Accordingly, brefeldin A, which is known to suppress Arf1 activation by binding to its upstream activator ArfGEFs, blocks the cytokine secretion from activated T cells^7,8^, raising the possibility that Arf1 plays an important role in cytokine secretion from activated mast cells as well. In addition, by using inhibitory peptide, Nishida et al. have shown that Arf1 regulates mast cell degranulation downstream of the Gab2-PI3K axis^9^. However, these findings rely on only pharmacological blockade of the Arf1 pathway, and in this sense physiological roles of Arf1 in mast cells still remain obscure.

To unmask the functional role of the Arf pathway in mast cell, we took advantage of tamoxifen-induced Arf1 deletion system, and found that the lack of Arf1 impairs mast cell proliferation upon IL-3 stimulation. On the other hand, contrary to our expectation, Arf1-deficiency had no impact on FcεRI-induced cytokine secretion. We also show that Arf1 is dispensable for mast cell degranulation.

## Results

### Loss of Arf1 attenuates mast cell expansion

**Figure 1:**
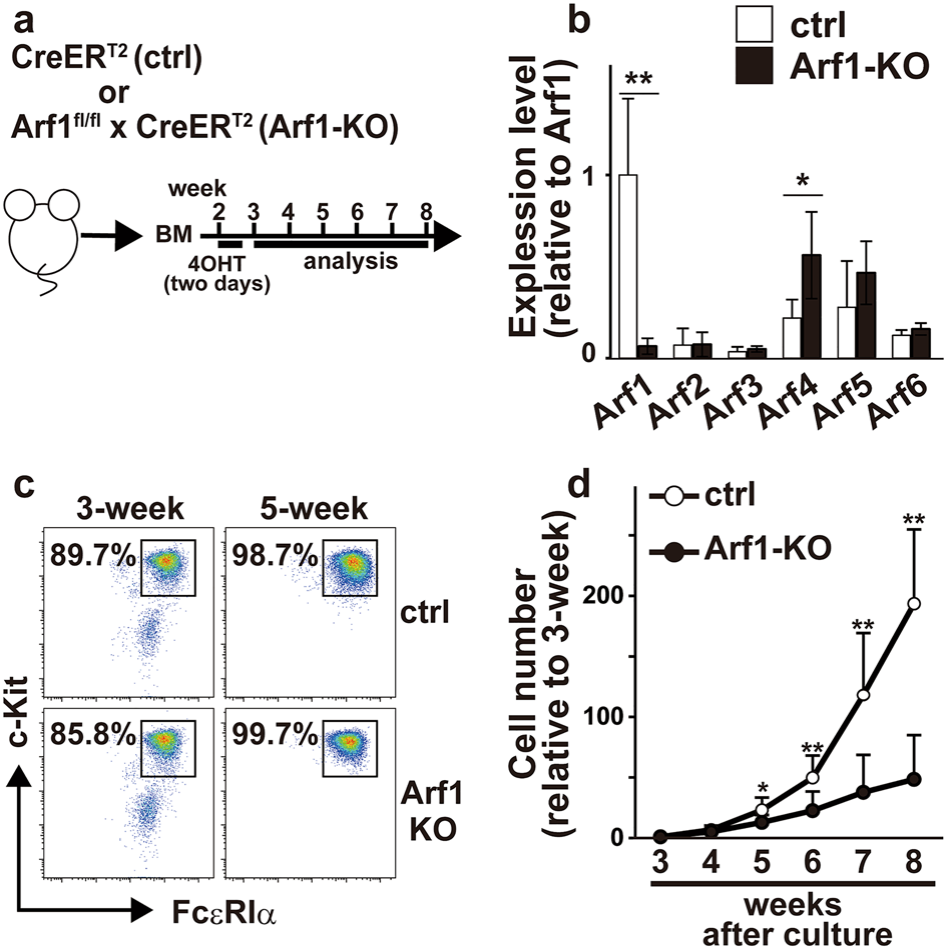
Characterization of Arf1-deficient BMMCs. (**a**) Schema of the BMMC culture. (**b**) Quantitative PCR (qPCR) analysis of expression levels of *Arf* family members relative to *Cyclophilin A* in BMMCs derived from control (ctrl) and Arf1-KO mice (n = 6, each). Shown are relative expression levels normalized to *Arf1* in control mice (mean ± SD). (**c**) Flow cytometric profiles of c-Kit^+^FcεRIα^+^ cells at 3- and 5-week after culture. Shown are representative of eight independent experiments. (**d**) The cell numbers of BMMCs relative to those at 3-week after culture (n = 8, each). Mean ± SD. *p < 0.05, **p < 0.01.

Since Arf1 was predominantly expressed in bone marrow-derived mast cells (BMMCs) (Fig. 1a,b), we focused on Arf1 among Arf family members to investigate the physiological roles of the Arf pathway in mast cells. We bred Arf1 conditional knockout (*Arf1*^fl/fl^) mice^10^ with *R26-*CreER^T2^ mice^11^ to generate *Arf1*^fl/fl^; *R26-*CreER^T2^ mice, where Arf1 can be efficiently deleted upon 4-hydroxytamoxifen (4-OHT) treatment. BMMCs were generated in vitro from the bone marrow cells from *R26-*CreER^T2^ mice (control) and *Arf1*^fl/fl^; *R26-*CreER^T2^ mice (Arf1-KO) after treatment with 4-OHT. Unfortunately, addition of 4-OHT from the beginning of culture caused robust cell death even in control cells (data not shown). Although the precise reason remains elusive, it may reflect the toxic effect of Cre expression in hematopoietic stem cells^12^. We therefore treated the cells with 4-OHT from day12 to day14 after starting culture to minimize non-specific effect of 4-OHT on BMMC generation (Fig. 1a). By using qPCR analysis, we confirmed that expression level of *Arf1* mRNA was virtually absent in Arf1-KO BMMCs whereas *Arf4* expression was slightly increased (Fig. 1b). Arf1-KO BMMCs expressed similar levels of FcεRI and c-Kit on their surface to control BMMCs, and there was little or no difference in maturation kinetics between control and Arf1-KO BMMCs as well (Fig. 1c and Supplemental Fig. 1). On the other hand, however, mast cell numbers were markedly decreased in the absence of Arf1 (Fig. 1d). It should also be noted that Arf1-deficient BMMCs showed slightly but significantly decreased FSC values, which indicates a reduction in cell size, compared with control BMMCs (Supplemental Fig. 1b). These data suggest that the Arf1 plays a critical role in mast cell expansion but not in mast cell maturation.

### Arf1 is dispensable for mast cell survival

**Figure 2:**
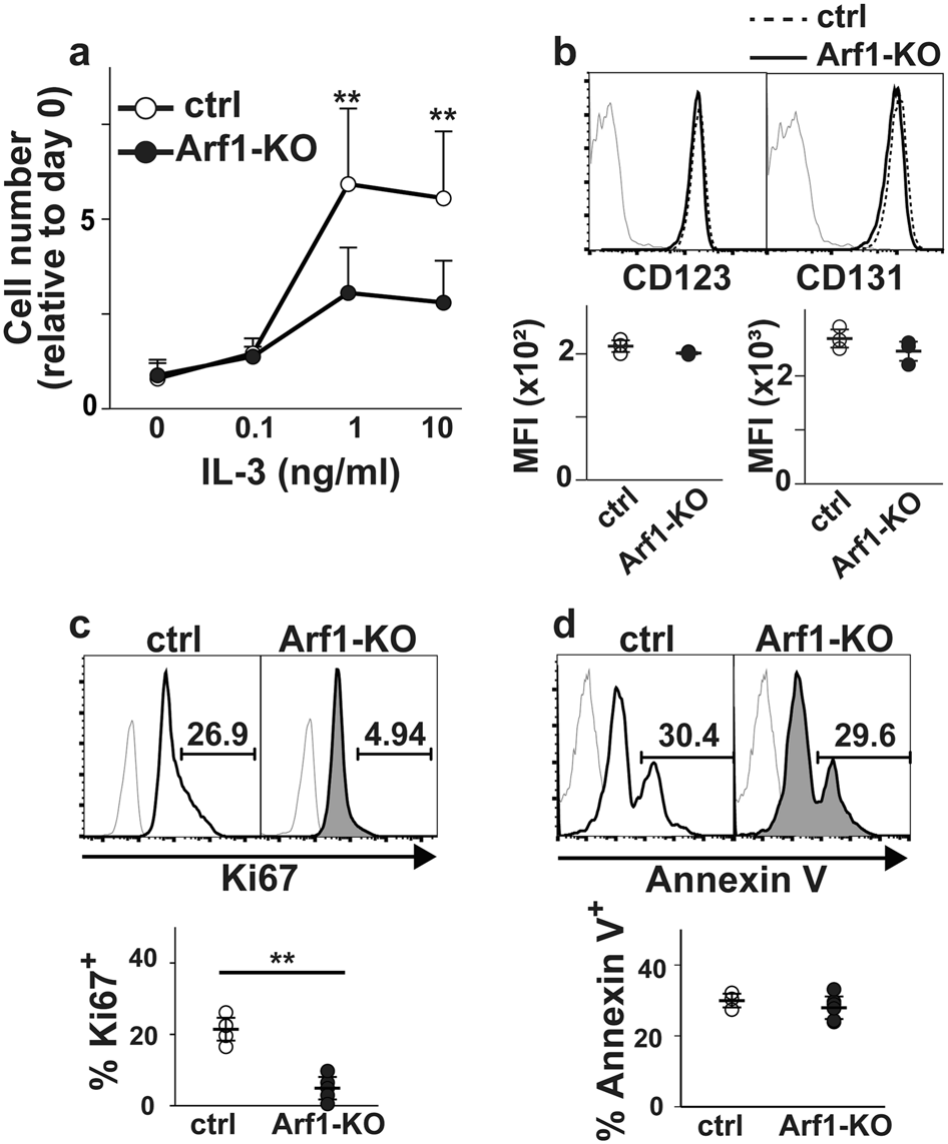
Cell proliferation is suppressed in Arf1-deficient BMMCs. (**a**) Either control (ctrl) or Arf1-KO BMMCs at 5-week after culture (n = 8, each) were stimulated with the indicated concentration of IL-3 for 5 days. The cell numbers were determined using Cell Counting Kit-8 and shown relative to the cell numbers at day 0 (mean ± SD). (**b**) Expression levels of CD123 and CD131 in BMMCs at 5-week after culture were evaluated by FACS (n = 3, each). Shown are representative FACS profiles (top) and mean fluorescent intensities (MFI) (bottom). Gray thin lines on FACS plot indicate negative stained signal with isotype controls. Mean ± SD. (**c**, **d**) The proportions of Ki67^+^ cells (**c**) and Annexin V^+^ cells (**d**) in BMMCs at 5-week after culture were evaluated by FACS (n = 4, each). Shown are representative FACS profiles (top) and proportions (bottom). Gray thin lines on FACS plot indicate negative stained signal with isotype controls (for Ki67) or control reagents (for Annexin V). Mean ± SD. **p < 0.01.

It should be noted that expansion of Arf1-KO BMMCs reached a plateau at around 1 ng/ml of IL-3, which was quite similar to control BMMCs (Fig. 2a), excluding the possibility that Arf1-KO BMMCs need a higher dose of IL-3 to respond. Consistently, we failed to detect any difference in expression levels of IL-3 receptor (IL-3R), which consists of IL-3Rα (CD123) subunit and βc (CD131) subunit^13^, between control and Arf1-KO BMMCs (Fig. 2b). One can argue that a defect in cell expansion is attributed to either impaired cell proliferation, enhanced cell death, or both. To distinguish these possibilities, we evaluated proportions of Ki67^+^ cells and Annexin V^+^ cells: the former reflects proliferative cell population while the latter reflects cells undergoing apoptosis. Given that the Arf pathway is required for survival but not proliferation of activated T cells^10^, it seems reasonable to speculate that Arf1-KO BMMCs exhibit enhanced apoptosis as well. Contrary to our expectation, however, we found that Arf1-deficiency had little or no impact on apoptosis during IL-3-induced mast cell expansion (Fig. 2d) whereas cell proliferation was markedly abrogated (Fig. 2c). These results indicate that the defect in expansion of Arf1-KO BMMCs resulted from impaired cell proliferation but not enhanced apoptosis.

### Arf1 facilitates mTORC1 activation, but not ERK, in BMMCs upon IL-3 stimulation

**Figure 3:**
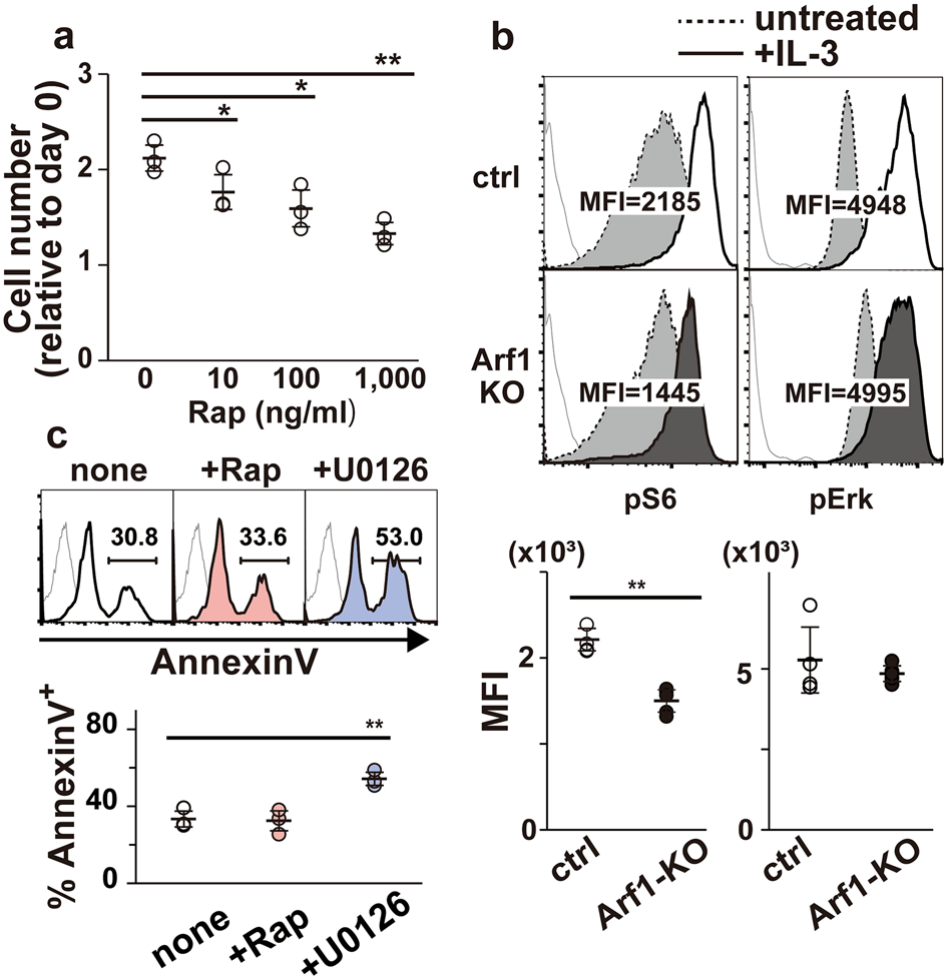
The mTORC1 signal is decreased in Arf1-deficient BMMCs. (**a**) Control BMMCs at 5-week after culture (n = 3) were stimulated with IL-3 (10 ng/ml) along with the indicated concentration of rapamycin (Rap) for 5 days. The cell numbers were determined using Cell Counting Kit-8 and shown relative to the cell numbers at day 0 (mean ± SD). (**b**) Either control (ctrl; n = 4) or Arf1-KO (n = 6) BMMCs were pretreated without IL-3 for 18 h, stimulated without (dotted line) or with (solid line) 10 ng/mL of IL-3 for 20 min, and assayed for pS6 (left) and pErk (right) signals by FACS. Shown are representative FACS profiles (top) and mean fluorescence intensities (MFI) (bottom). Gray thin lines on FACS plot indicate negative stained signal with isotype controls. Mean ± SD. (**c**) Control BMMCs depleted of Annexin V^+^ cells were cultured in the presence of 10 ng/mL IL-3 without (none) or with 0.1 µM of rapamycin (+ Rap) or 10 µM of U0126 (+ U0126) for 3 days (n = 3, each). The proportions of Annexin V^+^ cells were evaluated by FACS. Shown are representative FACS profiles (top) and proportions (bottom). Gray thin lines on FACS plot indicate negative stained signal with control reagents. Mean ± SD. *p < 0.05, **p < 0.01.

It is well-established that the mammalian target of rapamycin complex 1 (mTORC1) is a key regulator of cell proliferation^14^. Actually, treatment with rapamycin, a potent inhibitor of mTORC1, suppressed proliferation of BMMCs albeit with a relatively higher dose (Fig. 3a). We thus investigated whether Arf1-deficiency affects mTORC1 activation downstream of IL-3R. FACS analysis revealed that IL-3 signal-induced phosphorylation of S6 protein, a well-known target of the mTORC1-p70 S6K axis^14^, was significantly decreased in Arf1-KO BMMCs when compared with control BMMCs (Fig. 3b, left). On the other hand, there was little or no difference in phosphorylation status of ERK, another key signaling component downstream of IL-3R^13^, between control and Arf1-KO BMMCs upon IL-3 stimulation (Fig. 3b, right), suggesting that Arf1 is specifically involved in mTORC1 activation pathway. Essentially the same results were obtained with western blot analysis (Supplemental Fig. 2a). Since ERK activation is known to contribute to S6 phosphorylation via its downstream target p90 RSK^15^, we re-evaluated IL-3 signal-induced phosphorylation status of S6 in the presence of U0126, a potent inhibitor of the ERK pathway, confirming that Arf1 is required for optimal mTORC1 activation downstream of IL-3R (Supplemental Fig. 2b). This is in line with the finding that Arf1-deficiency led to a reduction of cell size of BMMCs^14^ (Supplemental Fig. 1b). Notably, rapamycin had no impact on mast cell survival while apoptosis was robustly induced in the presence of U0126 (Fig. 3c), also demonstrating that Arf1-deficiency in BMMCs is phenocopied by pharmacological blockade of the mTORC1 pathway.

Several reports have shown that phosphatidic acid, which is generated via phospholipase D (PLD), is required for mTORC1 activation^16,17^. Considering that PLD is one of Arf1 effectors^6^, we speculate that PLD contributes to the Arf1-mTORC1 axis downstream of IL-3R. We thus examined whether PLD inhibition affects IL-3 signal-induced S6 phosphorylation and proliferation. Treatment of BMMCs with PLD-specific inhibitor FIPI, however, had no impact on S6 phosphorylation or mast cell proliferation (Supplemental Fig. 2c,d). We further found that BFA, a potent inhibitor of ArfGEF1, failed to suppress S6 phosphorylation or proliferation upon IL-3 stimulation in BMMCs, raising the possibility that IL-3 signal-induced Arf1 activation is mediated by BFA-insensitive GEFs like cytohesins (Supplemental Fig. 2c,d).

### Loss of Arf1 has little effect on mast cell degranulation

**Figure 4:**
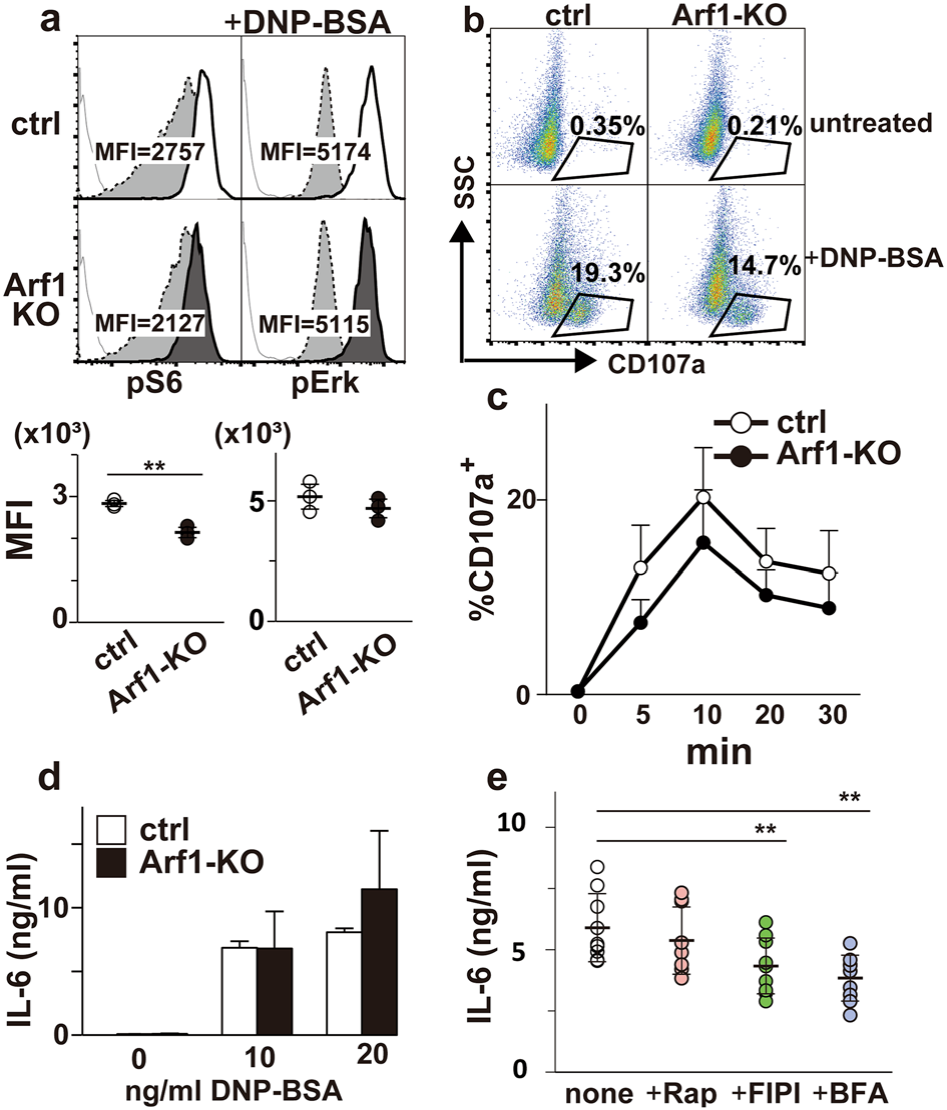
Degranulation reaction is normal in Arf1-deficient BMMCs. (**a**) Either control (ctrl; n = 3) or Arf1-KO (n = 3) BMMCs at 5-week after culture sensitized with anti-DNP IgE were stimulated without (dotted line) or with (solid line) 20 ng/mL of DNP-BSA for 20 min and evaluated for pS6 (left) and pErk (right) signals by FACS. Shown are representative FACS profiles (top) and mean fluorescence intensities (MFI) (bottom). Gray thin lines on FACS plot indicate negative stained signal with isotype controls. Mean ± SD. (**b**) Control (ctrl) or Arf1-KO BMMCs sensitized with anti-DNP IgE were stimulated without (untreated) or with (+ DNA-BSA) 20 ng/mL of DNP-BSA for 20 min. Shown are FACS profiles and proportion of CD107a^+^ cells representative of four. (**c**) Proportions of CD107a^+^ cells in control (ctrl) or Arf1-KO BMMCs stimulated as in (**b**) for the indicated time periods were evaluated by FACS (n = 4, each). (**d**) Control (ctrl) or Arf1-KO BMMCs sensitized with anti-DNP IgE were stimulated with the indicated concentrations of DNP-BSA for 24 h (**d**; n = 5, each). Concentrations of IL-6 in the culture supernatants were then quantified by ELISA. (**e**) Control BMMCs sensitized with anti-DNP IgE were stimulated with 20 ng/mL of DNP-BSA along without (none) or with 0.1 µM of rapamycin (+ Rap), 0.1 µg/mL of FIPI (+ FIPI), or 0.1 µM of BFA (+ BFA) for 24 h (e; n = 9, each). Concentrations of IL-6 in the culture supernatants were then quantified by ELISA. **p < 0.01.

Given that FcεRI plays a critical role in mast cell activation, we examined whether FcεRI-mediated signaling events in BMMCs are affected in the absence of Arf1. BMMCs sensitized with anti-DNP IgE were stimulated with DNP-BSA, and were subjected to FACS analysis. Interestingly, like IL-3R, FcεRI-induced S6 phopshorylation was attenuated in Arf1-KO BMMCs while ERK activation was intact (Fig. 4a). We further found that Akt activation upon FcεRI stimuli was partly attenuated in Arf1-KO BMMCs as well (Supplemental Fig. 3a), raising the possibility that Arf1 regulates the PI3K/mTORC1 pathway downstream of FcεRI. Essentially the same results were obtained when BMMCs were stimulated with stem cell factor (SCF), the c-Kit ligand (Supplemental Fig. 3b). These results strongly suggest that the Arf1 pathway in BMMCs is generally involved in mTORC1 activation irrespective of input signals.

Pioneering study by Kim et al*.* have clearly demonstrated that pharmacological blockade of mTORC1 has little impact on FcεRI-mediated mast cell activation^18^. We therefore investigated whether Arf1-deficiency perturbs mast cell activation upon FcεRI stimulation. In marked contrast to the previous report demonstrating the important role of Arf1 during FcεRI-induced degranulation^9^, we failed to detect any significant difference in CD107a cell surface expression, a measure of granule exocytosis, between control and Arf1-KO BMMCs albeit with slightly reduced trend in Arf1-KO (Fig. 4b,c). Essentially the same results were obtained with a classical degranulation assay (Supplemental Fig. 3c), also suggesting that Arf1-deficiency has no impact on mast cell granular component release. To further examine a role of Arf1 in FcεRI-induced cytokine secretion, supernatants of activated BMMCs were collected and assayed for IL-6 production. We found that Arf1-KO BMMCs secreted IL-6 upon FcεRI stimulation to a level comparable to control BMMCs under conditions where FIPI or BFA attenuated IL-6 secretion (Fig. 4d,e). It is of interest to note that neither BFA nor FIPI affects FcεRI-induced S6 phosphorylation or degranulation (Supplemental Fig. 3d,e). These data clearly indicate that Arf1 is dispensable for FcεRI-mediated mast cell activation except for mTORC1 signal, and that Arf1 in BMMCs functions independently from either the BFA-sensitive pathway or the FIPI-sensitive pathway.

## Discussion

Mast cell numbers are shaped by two growth factors SCF and IL-3: the former is required for development and maintenance of mast cells under steady-state conditions while the latter plays a critical role in expansion of mast cells under allergic conditions like parasite infection^19^. Accordingly, it is well-established that mouse mast cells can differentiate from bone marrow cells in response to IL-3 (mucosal tissue type) or IL-3 along with SCF (connective tissue type) in vitro^20^. Here we demonstrated for the first time that Arf1 facilitates mast cell proliferation upon IL-3 stimulation via mTORC1 signal, raising the possibility that a therapeutic approach against Arf1 is beneficial to reducing mast cell expansion associated with inflammation. We also found that Arf1-deficiency attenuated SCF-induced S6 phosphorylation, which presumably reflects the blockade of mTORC1 signal as well. Given that c-Kit, a receptor for SCF, has been implicated in the pathogenesis of several neoplastic diseases including mastocytosis^21^, Arf1 inhibitor could also be applicable to mast cell proliferative disorders like Kit-mediated tumorigenesis. Consistently, rapamycin has been investigated as a potential approach to block proliferating mast cells^22^.

Multiple environmental inputs including amino acids, stress, growth factors, and energy status regulate mTORC1 activity in order to control cell growth^23,24^. Although PLD, a downstream effector of Arf1, has been implicated to regulate mTORC1 signal^16,17^, a potent PLD inhibitor FIPI had little or no impact on mTORC1 signal in mast cells (Supplemental Figs. 2 and 3) while FIPI substantially attenuated FcεRI-induced IL-6 production (Fig. 4e). On the other hand, recent reports have revealed a novel role of Arf1 in mTORC1 activation on the amino acid sensing pathway^25–27^. Intriguingly, Arf1 plays an important role in the regulation of mTORC1 by glutamine and asparagine while the well-characterized Rag GTPase pathway mediates mTORC1 activation downstream of leucine, arginine, methionine, and some other amino acids^28–34^. Our finding that the lack of Arf1 causes only marginal blockade of mTORC1 signal in mast cells may reflect existence of such distinct signaling cascades and mechanisms. In contrast to mTORC1 signal, we found little or no impact on ERK signal in the absence of Arf1 during BMMC activation (Figs. 3b and 4a, and Supplemental Fig. 3b). It is of interest to note, however, that basal ERK signal without IL-3 (Fig. 3b and Supplemental Fig. 3b), but not with IL-3 (Fig. 4a), reproducibly increased in Arf1-KO BMMCs compared to control (data not shown). Although its precise reason remains obscure, it may reflect some compensatory mechanism to maintain mast cell survival in the absence of Arf1.

Since Arf1 has been reported to control membrane traffic and organelle structure, it seems reasonable to speculate that Arf1 is essential for vesicle transport like mast cell degranulation and cytokine secretion. Actually, Nishida et al. have previously demonstrated that Arf1 is required for FcεRI-induced granule translocation in BMMCs^9^. On the contrary to our expectation, however, Arf1-deficiency failed to cause a significant defect in mast cell degranulation (Fig. 4b,c). Although the precise reason for this discrepancy remains unknown, we should note that the conclusion by Nishida et al. relies on observations obtained by using “Arf1-inhibitory peptide” which is derived from N-terminal portion of Arf1 or siRNA against *Arf1*. Considering that Arf proteins show relatively high homology among family members, it could be possible that perturbation of other Arf family members but not Arf1 leads to the blockade of mast cell degranulation. Alternatively, one can speculate that Arf1-deficiency in mast cells is compensated with other family members such as Arf4 (Fig. 1b). However, since germ-line knockout of *Arf4* gene results in embryonic lethality^35^ and there is no report regarding mast cell-specific deletion of *Arf4* at present, we cannot formally exclude a possible contribution of Arf4 to mast cell degranulation. We also found that the lack of Arf1 had no effect on FcεRI-induced IL-6 secretion. It is of interest to note that cytokine secretion remains intact in T cells lacking both Arf1 and Arf6 as well^10^. Future studies will be needed to clarify the precise mechanism underlying FcεRI-induced granule translocation.

## Methods

### Mice

All experimental procedures involving animals were approved by the Guideline for Animal Experimentation, Kansai Medical University (Approval number: 22-089(2)). All mouse strains used in this study were backcrossed to C57BL/6 for at least nine generations. The floxed *Arf*1 (*Arf1*^fl/fl^) mouse line was established^10^ and crossed with *R26-*CreER^T2^ mice^11^. Age-matched *Arf1*^+/+^: *R26*-CreER^T2^ and *Arf1*^fl/+^: *R26*-CreER^T2^ mice were used as a control. We used male and female mice aged between 7 and 18 weeks in all experiments unless otherwise stated. All mice were maintained in a specific pathogen-free facility and used according to our institutional guidelines. The study is reported in accordance with ARRIVE guidelines.

### BMMC differentiation

Bone marrow cells (1 × 10^5^ cells/ml) were cultured (37 °C, 5% CO_2_) as a single cell suspension in culture medium (RPMI 1640 (Fujifilm) supplemented with 10% FCS (HyClone), 55 µM 2-ME, 100 U/mL penicillin, 100 mg/mL streptomycin, non-essential amino acids, 1 mM sodium pyruvate, 10 mM HEPES, and 10 ng/mL mouse IL-3 (BioLegend)) by 9 weeks. From day 12 to day 14, the cells were treated with 1 µM of 4-OHT (Sigma). We confirmed that more than 98% of the cells were c-Kit^+^FcεRIα^+^ for 5-week culture.

### Quantitative PCR

Total RNA was extracted using RNeasy Micro Kit (QIAGEN), followed by PrimeScript RT Master Mix (Takara) reaction. THUNDERBIRD SYBR qPCR Mix (Toyobo) was used to evaluate gene expression on a Rotor-Gene Q (QIAGEN). Primers used were as follows: *Arf1*, 5′-GTTTGCCAACAAGCAGGAC-3′ and 5′-TGGCCTGAATGTACCAGTTC-3′; *Arf2*, 5′-CGAGTAGCGTTTTCGTGAG-3′ and 5′-TCAAAGACATTCCCCATTGTAG-3′; *Arf3*, 5′-GAAACTCGGGGAGATTGTCA-3′ and 5′-GTCCCAGACTGTGAAGCTGAT-3′; *Arf4*, 5′-TTCACAGTATGGGATGTTGGTGGTCA-3′ and 5′-GCACAGCTGCTCCTTCCTGGATT-3′; *Arf5*, 5′-AGTCTGCTGATGAACTCCAGAA-3′ and 5′-GCTTGTTGGCAAACACCA-3′; *Arf6*, 5′-TCCTAATGAGCGTCCTCCAC-3′ and 5′-TCCTAGGAATGGGTTTTGGA-3′; and *Cyclophilin A*, 5′-ATGGCACTGGCGGCAGGTCC-3’ and 5′-TTGCCATTCCTGGACCCAAA-3′.

### Cell proliferation assay

To deplete IL-3, BMMCs were incubated in RPMI1640 with the supplements in the absence of IL-3 for 18 h. Ten thousand cells were then seeded onto 96-well plate and cultured for 5 days with the indicated concentrations of IL-3. Cell numbers were determined using a Cell Counting Kit-8 according to the manufacturer's instructions (Dojindo Laboratories).

### Abs

Abs used in FACS analysis were as follows: CD131 (REA193) from Miltenyi Biotec; FcεRIα (MAR-1) and Ki67 (SolA15) from eBioscience; phospho-S6 ribosomal protein (Ser235/236) (D57.2.2E) and phospho-p44/42 MAPK (Erk1/2) (Thr202/Tyr204) (197G2) from Cell Signaling Technology; CD117 (c-Kit) (ACK2), Annexin V, CD123 (5B11) and CD107a (LAMP-1) (1D4B) from BioLegend. Isotype control Abs in FACS analysis were as follows: recombinant human IgG1 (REA293) from Miltenyi Biotec; rabbit IgG (DA1E) from Cell Signaling Technology; rat IgG2a (RTK2758), rat IgG2b (RTK4530), and Armenian hamster IgG (HTK888) from BioLegend. Alexa Fluoro 647-conjugated BSA (Thermo Fisher) was used as a control reagent for Annexin V staining.

### Flowcytometry

Isolated cells were stained with the appropriate Abs along with 7-aminoactinomycin D (7-AAD; Sigma). For intracellular staining of Ki67, cells were treated with Cytofix/Cytoperm (BD Biosciences). To assess the impact of inhibitors on cell survival, after removal of Annexin V^+^ cells by MojoSort (BioLegend), BMMCs were cultured for 3 days with the indicated inhibitors and then proportions of Annexin V^+^ cells were evaluated by FACS analysis according to the manufacturer's instructions (BioLegend). For detection of phosphorylated proteins, stimulated cells were immediately fixed with Phosflow Lyse/Fix buffer (BD Biosciences), followed by permeabilization with Perm buffer III (BD Biosciences) according to the manufacturer's instructions. Data were acquired on a FACSCanto II (BD Biosciences) or an Attune NxT Flow Cytometer (Thermo Fisher Scientific) and analyzed with FlowJo Software (BD Biosciences).

### Western blot analysis

After stimulation, cells were washed once with ice-cold PBS, and lysed in a lysis buffer solution (20 mM Tris–HCl, pH 7.5, 2 mM EGTA, 25 mM β-glycerophosphate, 1% Triton X-100, 2 mM dithiothreitol, 1 mM vanadate, 1 mM phenylmethylsulfonyl fluoride and 1% aprotinin), followed by centrifugation at 15,000 × *g* for 5 min. The supernatant mixed with Laemmli sample buffer solution was run on a Bolt 4–12% Bis–Tris Plus gel (Invitrogen), transferred to PVDF membrane (Trans-blot Turbo Transfer Pack, Bio-Rad), and subjected to western blot analysis against phospho-ERK (Thr202/Tyr204) (E10, CST), phosphor-S6 (Ser235/236) (D57.2.2E, CST), phosphor-Akt (Ser473) (D9E, CST), or αtubulin (11H10, CST) as previously described^10^. Western Lightning Plus-ECL (PerkinElmer) was used for the detection of chemiluminescence, followed by analysis on a FUSION SOLO imaging system (M&S Instruments) to obtain and quantify digital images.

### Measurement of degranulation

BMMCs were sensitized with 1 µg/mL of anti-DNP IgE (SPE-7, Sigma) in culture medium for 18 h, and then treated with 20 ng/mL of DNP-BSA for 20 min to activate them. Degranulation of BMMCs was assessed by flow cytometric measurement of the surface expression of CD107a (Lamp1). In some experiments, degranulation was evaluated by measuring the release of β-hexosaminidase upon stimulation as well. After washing with Tyrode’s buffer solution (10 mM HEPES, 130 mM NaCl, 6.2 mM D-Glucose, 3.0 mM KCl, 1.4 mM CaCl_2_, 1.0 mM MgCl_2_ and 0.1% BSA), sensitized BMMCs (1 × 10^5^ cells in 100 µL of Tyrode’s buffer solution/well in 96-well plate) were stimulated with DNP-BSA for 30 min, and then 50 µL of supernatants were incubated for 60 min with equal volume of 1 mM *p*-nitrophenyl-N-acetyl-p-d-glucosamine (Sigma) in 0.1 M sodium citrate buffer (pH 4.5) at 37 °C. The reaction was terminated by the addition of 0.2 M glycine (pH 10.7) and the released product, 4-*p*-nitrophenol, was detected by absorbance at 405 nm. The degranulation value was calculated by dividing 4-*p*-nitrophenol absorbance in the supernatant by the absorbance in detergent-solubilized unstimulated cell pellet.

### Measurement of cytokines

Sensitized BMMCs (1 × 10^4^ cells/well in 96-well plate) were stimulated with the indicated concentrations of DNP-BSA for 24 h, and concentrations of IL-6 in culture supernatants were detected with ELISA Max Deluxe (BioLegend).

### Statistical analysis

Unless otherwise indicated, statistical analysis was performed using unpaired Student’s *t* test. A *p* value < 0.05 was considered statistically significant.

## Supplementary Information

Supplementary Figures.

## Data Availability

The *Arf1*^fl/fl^ mouse line was deposited in RIKEN (accession no. CDB1027K; http://www2.clst.riken.jp/arg/micelist.html). All generated raw data and/or analyzed data from the current study can be available from the corresponding author on reasonable request.
