# Supplementary material for: Arf1 facilitates mast cell proliferation via the mTORC1 pathway
Source: Sci Rep. 2022 Dec 24;12:22297. doi: 10.1038/s41598-022-26925-1 (PMC9789986; doi:10.1038/s41598-022-26925-1)
Supplement: Supplementary file 1 — Supplementary Figures. [file 41598_2022_26925_MOESM1_ESM.pdf]

Arf1 facilitates mast cell proliferation via the mTORC1 pathway

Yui Kotani[1, 2], Mami Sumiyoshi[1], Megumi Sasada[2], Toshio Watanabe[2, *], and Satoshi Matsuda[1, *]

[1]Department of Cell Signaling, Institute of Biomedical Science, Kansai Medical University, Hirakata, Osaka 573-1010, Japan.

[2]Department of Biological Science, Graduate School of Humanities and Sciences, Nara Women's University, Nara 630-8506, Japan.

*E-mail address: matsudsa@hirakata.kmu.ac.jp or toshiwatana@cc.nara-wu.ac.jp

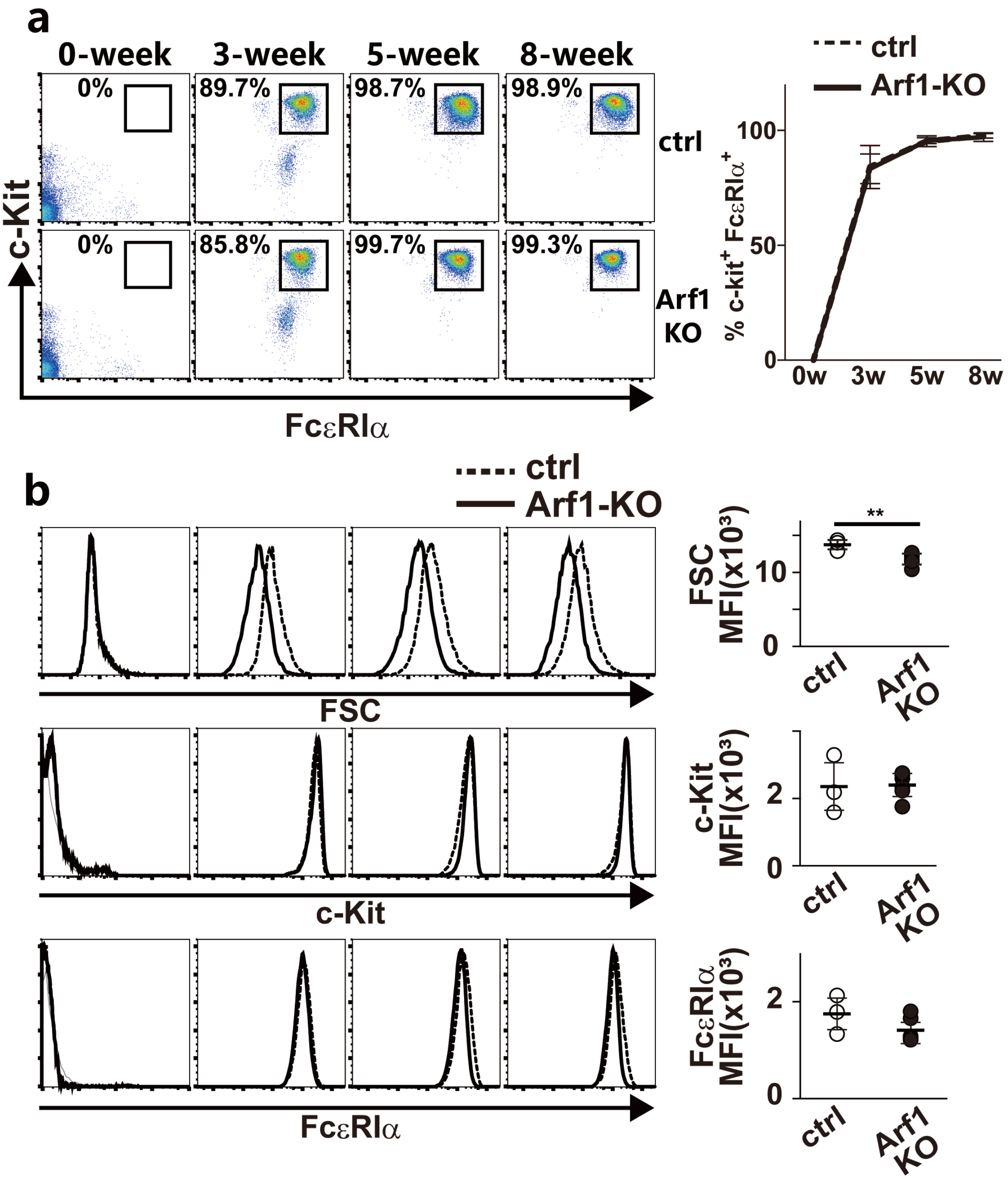

Supplemental Figure 1. FACS analysis of Arf1-deficient BMMCs.

(a) FACS profiles of c-Kit^{+}FcεRIα^{+} cells at the indicated time points after culture (control (ctrl); n=3, Arf1-KO; n=6). Shown are representative FACS profiles (left) and proportions

(right). Mean ± SD. (b) FSC values and expression levels of c-Kit and FcεRIα gated on c-Kit^{+}FcεRIα^{+} cells at the indicated time points after culture (control (ctrl); n=3, Arf1-KO; n=6). Shown are representative FACS profiles (left) and mean fluorescence intensities (MFI) at 5-week after culture (right). Gray thin lines on c-Kit and FcεRIα FACS plot at 0-week culture indicate negative stained signal with isotype controls. Mean ± SD. $^{**}p < 0.01$.

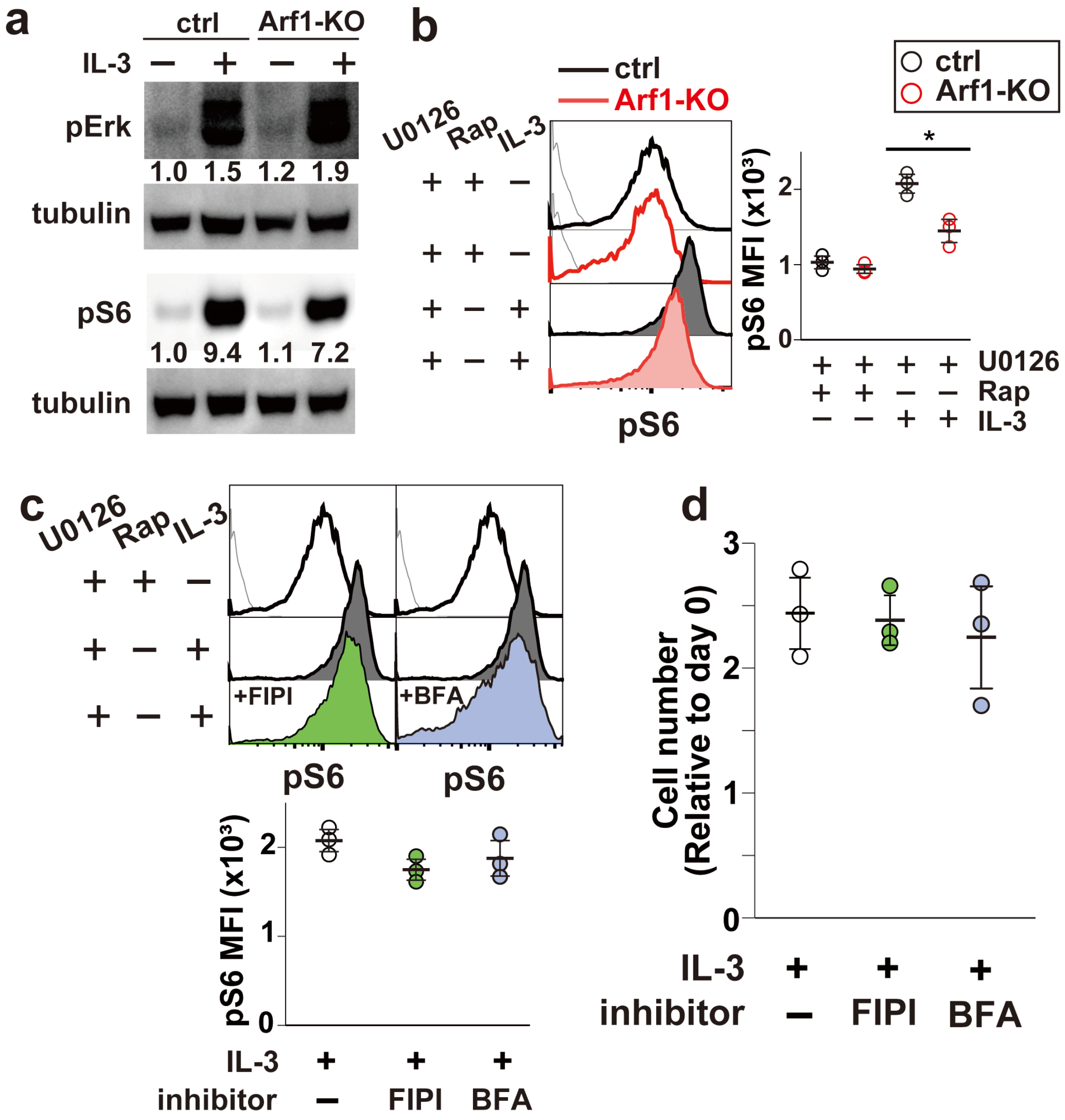

Supplemental Figure 2. FACS analysis of mTORC1 signal.

(a) Either control (ctrl) or Arf1-KO BMMCs at 5-week after culture were pretreated without IL-3 for 18 h, followed by stimulation with 10 ng/mL IL-3 for 20 min. The cell lysates were subjected to western blot analysis with the indicated antibodies, and analyzed

on a FUSION (M&S Instruments). Indicated are the signal intensities normalized to αtubulin. Values are expressed as fold increase to that of control BMMCs without stimulation (-). Data are representative of two independent experiments with similar results. Original blots are presented in Supplemental Figures 4 (pERK and pS6), 5 (pERK, long exposure) and 6 (tubulin). (b) Either control (ctrl) or Arf1-KO BMMCs at 5-week after culture were stimulated with (+) or without (-) IL-3 along with U0126 and/or rapamycin (+/-Rap) for 20 min (n=3, each) and evaluated for pS6 signal by FACS. Shown are representative FACS profiles (left) and mean fluorescence intensities (MFI) (right). Gray thin lines on FACS plot (top and middle upper) indicate negative stained signal with isotype controls. Mean ± SD. (c) Control BMMCs at 5-week after culture (n=3) were stimulated with (+) or without (-) IL-3 along with 0.1 μM of BFA (right) or 0.1 μg/mL of FIPI (left) for 20 min and evaluated for pS6 signal by FACS. Shown are representative FACS profiles (top) and mean fluorescence intensities (MFI) (bottom). Gray thin lines on FACS plot (top) indicate negative stained signal with isotype controls. Mean ± SD. (d) Control BMMCs at 5-week after culture (n=3) were stimulated with IL-3 along with 0.1 μM of BFA or 0.1 μg/mL of FIPI for 5 days. The cell numbers were determined using Cell Counting Kit-8 and shown as relative to the cell numbers at day 0 (mean ± SD). *$p<0.05$.

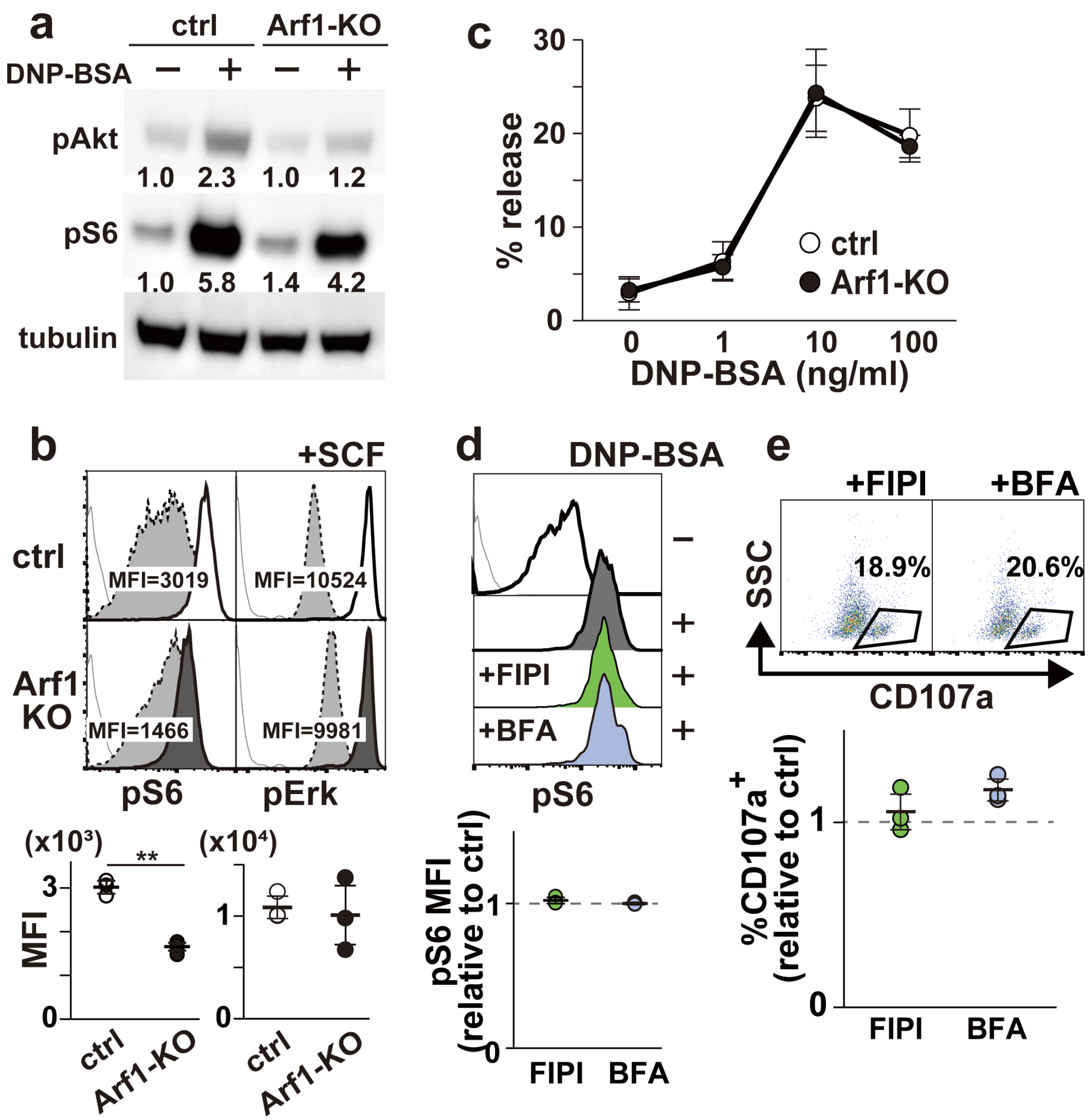

Supplemental Figure 3. Either BFA or FIPI has little effect on mast cell degranulation.

(a) Either control (ctrl) or Arf1-KO BMMCs at 8-week after culture were sensitized with anti-DNP IgE (SPE-7), followed by stimulation with 20 ng/mL DNP-BSA for 20 min. The cell lysates were subjected to western blot analysis with the indicated antibodies, and analyzed on a FUSION (M&S Instruments). Indicated are the signal intensities normalized

to αtubulin. Values are expressed as fold increase to that of control BMMCs without stimulation (-). Data are representative of two independent experiments with similar results. Original blots are presented in Supplemental Figures 7 (pAkt along with pS6) and 8 (tubulin). (b) Either control (ctrl; n=3) or Arf1-KO (n=3) BMMCs at 5-week after culture were cultured without IL-3 for 18 h, stimulated without (dotted line) or with (solid line) 100 ng/ml of stem cell factor (SCF) for 10 min, and assayed for pS6 (left) and pErk (right) signals by FACS. Gray thin lines on FACS plot indicate negative stained signal with isotype controls. (c) Either control (n=3, open circles) or Arf1-KO (n=3, closed circles) BMMCs at 8-week after culture were sensitized with anti-DNP IgE (SPE-7), and then β-hexosaminidase release was evaluated at 30 min after exposure to the indicated concentrations of DNP-BSA. Mean ± SD. Data are representative of two independent experiments with similar results. (d and e) Flow cytometric profiles of $CD107a^+$ cells of BMMCs, which were sensitized with anti-DNP IgE followed by stimulation with DNP-BSA along with the indicated inhibitors for 20 minutes. Shown are representative FACS profiles and mean fluorescent intensities (MFI) of pS6 signal (d) or proportions of $CD107a^+$ cells (e) relative to untreated control BMMCs (ctrl) (n=3, each). Gray thin line on FACS plot indicates negative stained signal with an isotype control. Mean ± SD. $^{**}p < 0.01$.

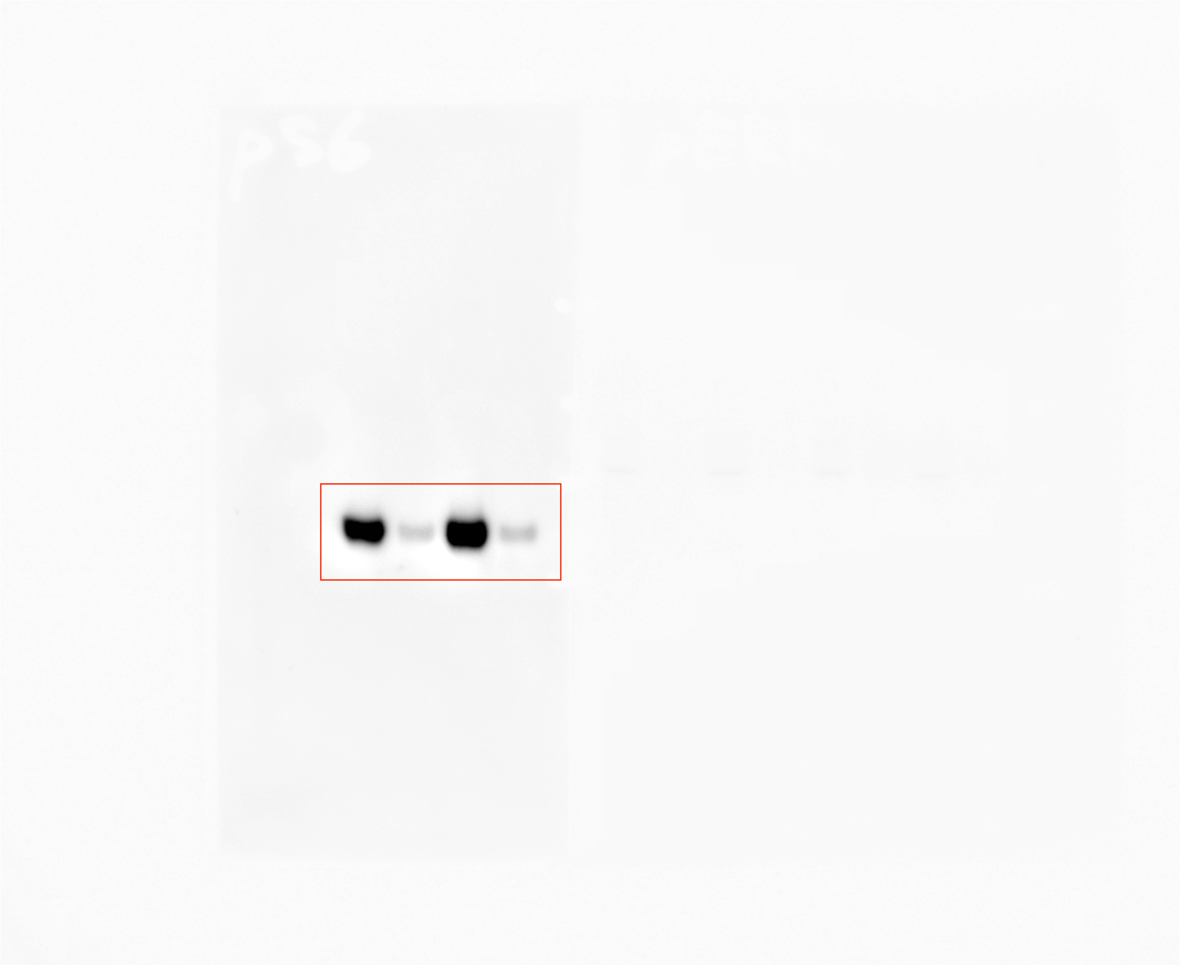

Supplemental Figure 4. Original blot for pS6 and pERK upon IL-3 stimulation.

A Bolt 4-12% Bis-Tris Plus gel with 15-well was run with samples as follows (from the right to left): lane 1, marker; lanes 2-5, negative and positive controls; lane 6, control BMMC without stimulation; lane 7, control BMMC with IL-3 stimulation; lane 8, KO BMMC without stimulation; lane 9, KO BMMC with IL-3 stimulation; lane 10, marker; lane 11, control BMMC without stimulation; lane 12, control BMMC with IL-3 stimulation; lane 13, KO BMMC without stimulation; lane 14, KO BMMC with IL-3 stimulation; lane 15, empty. The samples were then transferred to PVDF membrane, cut

into two parts at lane 10, and the right side part was subjected to western blot analysis against phospho-ERK (Thr202/Tyr204) while the left side part was subjected to western blot analysis against phospho-S6 (Ser235/236). Since pERK signals (the right side part) were relatively weak though a faint band can be still visible, the image containing lanes 11-14 (red box) alone was cropped, horizontally flipped, and indicated as pS6 signal in Supplemental Figure 2a, middle lower panel. Please note that the image of pERK signals with a long exposure time is presented in Supplemental Figure 5.

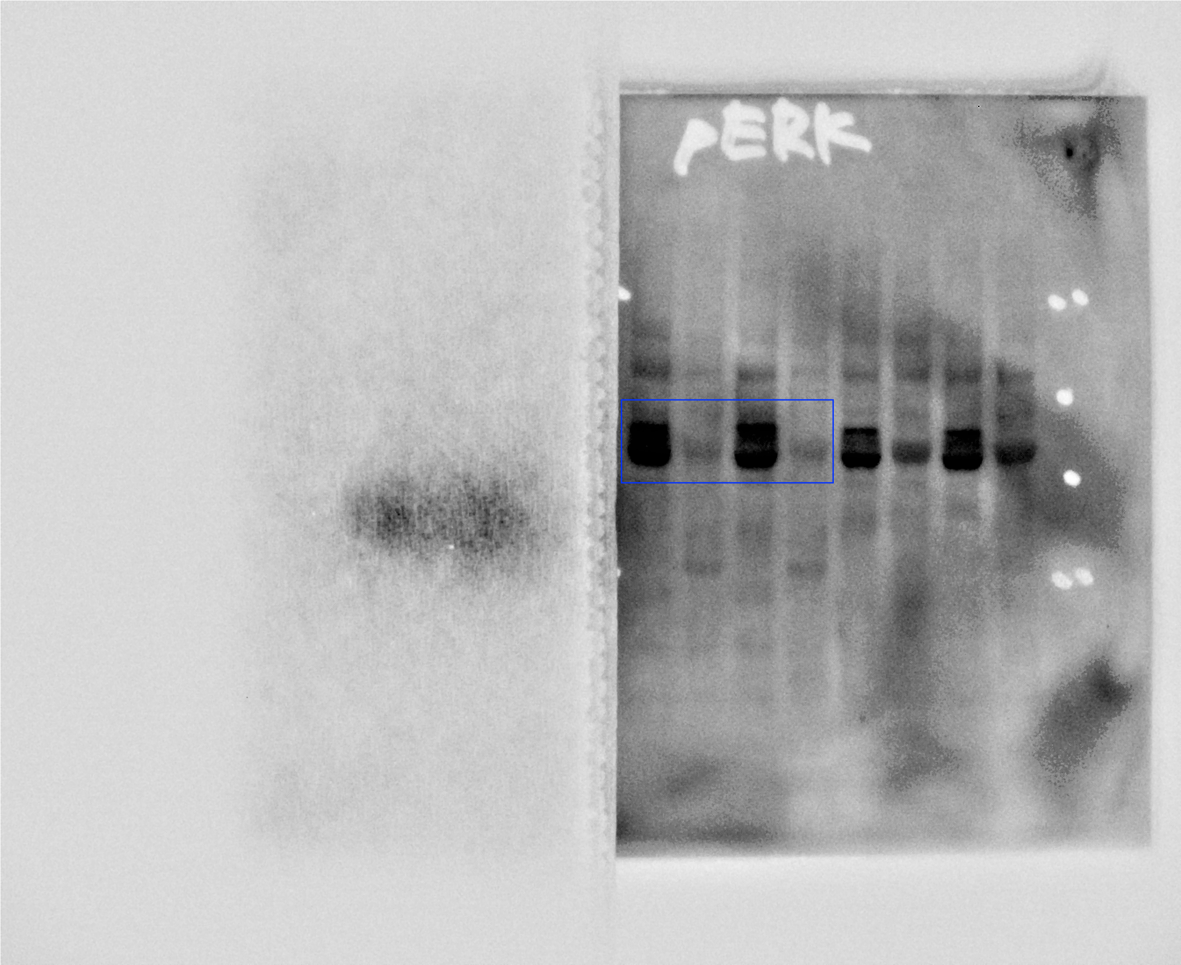

Supplemental Figure 5. Original blot for pERK with long exposure upon IL-3 stimulation. After the left side part (pS6 blot signal) was masked with sheets of paper to avoid saturation, pERK signal was acquired by using PVDF membrane as in Supplemental Figure 4 with a long exposure time. The image containing lanes 6-9 (blue box) was cropped, horizontally flipped, and indicated as pERK signal in Supplemental Figure 2a, top panel.

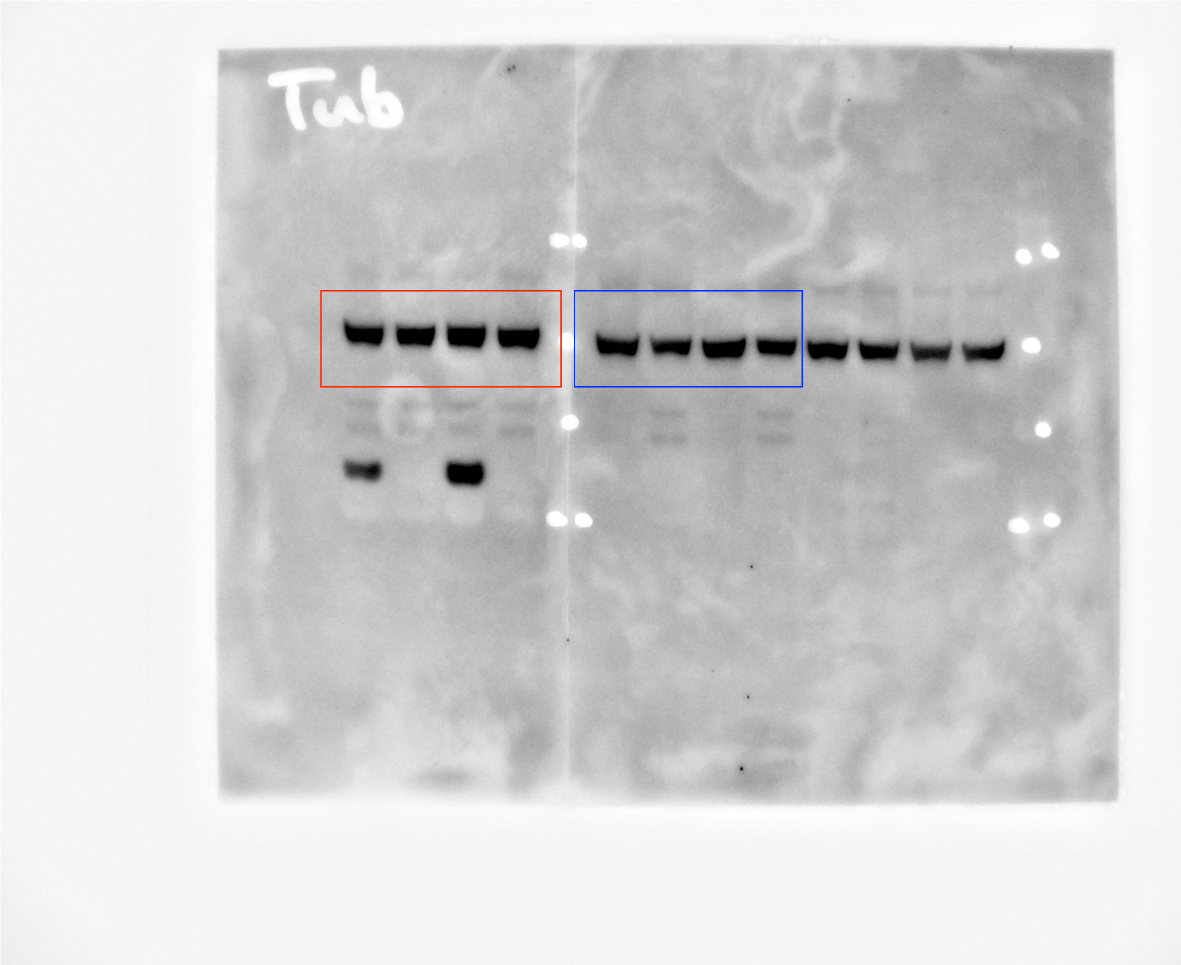

Supplemental Figure 6. Original blot for tubulin upon IL-3 stimulation.

PVDF membrane used in Supplemental Figures 4 and 5 was stripped and subjected to western blot analysis against αtubulin. The images containing lanes 6-9 (blue box) and lanes 11-14 (red box) were cropped, horizontally flipped, and indicated as tubulin signal in Supplemental Figure 2a, middle upper panel and bottom panel, respectively. Please note that pS6 signal partly remains in the left part of the membrane.

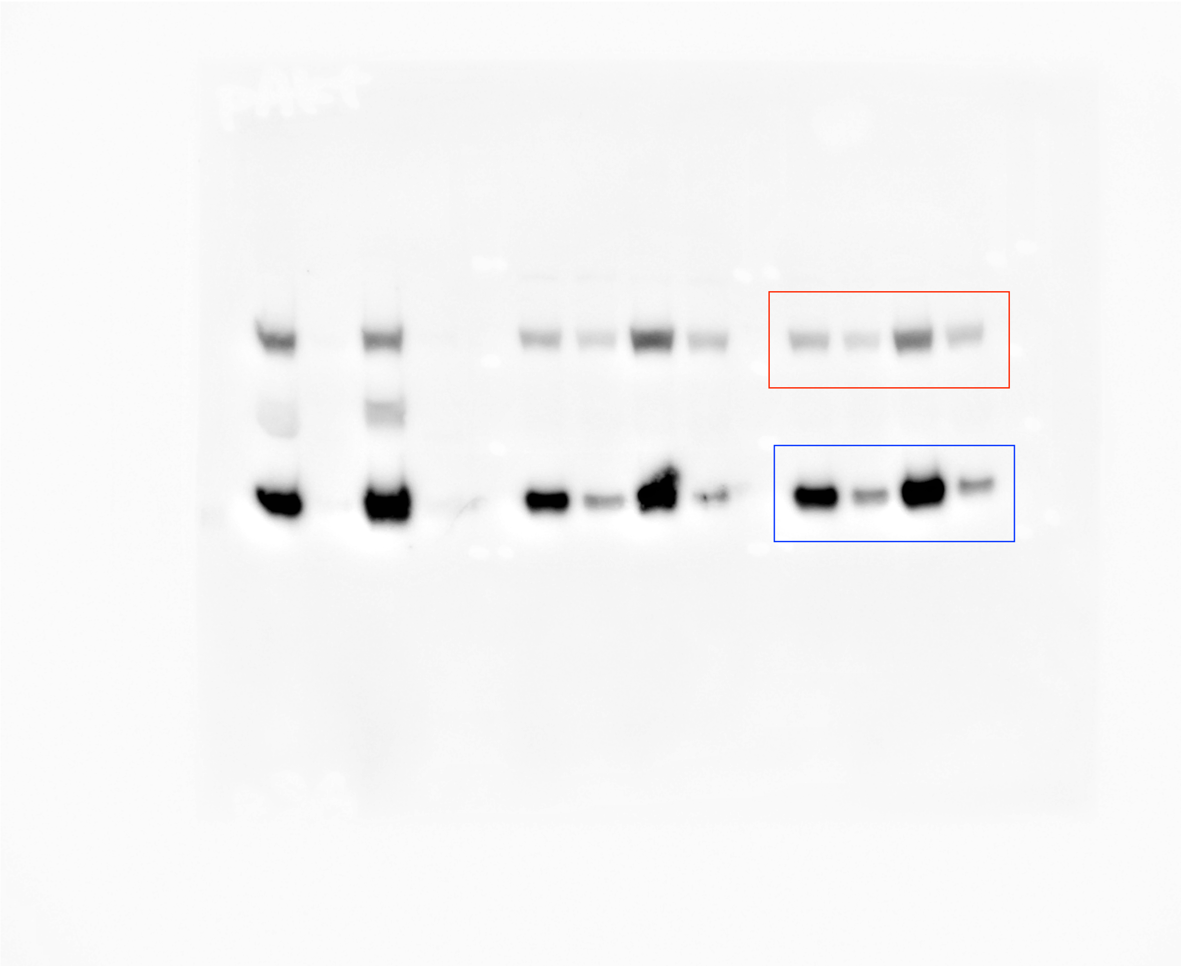

Supplemental Figure 7. Original blot for pAkt and pS6 upon FcεRI stimulation.

A Bolt 4-12% Bis-Tris Plus gel with 15-well was run with samples as follows (from the right to left): lane 1, marker; lane 2, control BMMC without stimulation (experiment#1); lane 3, control BMMC with stimulation (experiment#1); lane 4, KO BMMC without stimulation (experiment#1); lane 5, KO BMMC with stimulation (experiment#1); lane 6, marker; lane 7, control BMMC without stimulation (experiment#2); lane 8, control BMMC with stimulation (experiment#2); lane 9, KO BMMC without stimulation (experiment#2); lane 10, KO BMMC with stimulation (experiment#2); lanes 11-15, negative and positive

controls. The samples were then transferred to PVDF membrane, cut into two parts at approximately 37 kDa, and the upper part was subjected to western blot analysis against phospho-Akt (Ser473) while the lower part was subjected to western blot analysis against phospho-S6 (Ser235/236). The images containing lanes 2-5 (corresponding to experiment#1; red box for pAkt, blue box for pS6) were cropped, horizontally flipped, and indicated as pAkt and pS6 signals in Supplemental Figure 3a, top panel and middle panel, respectively.

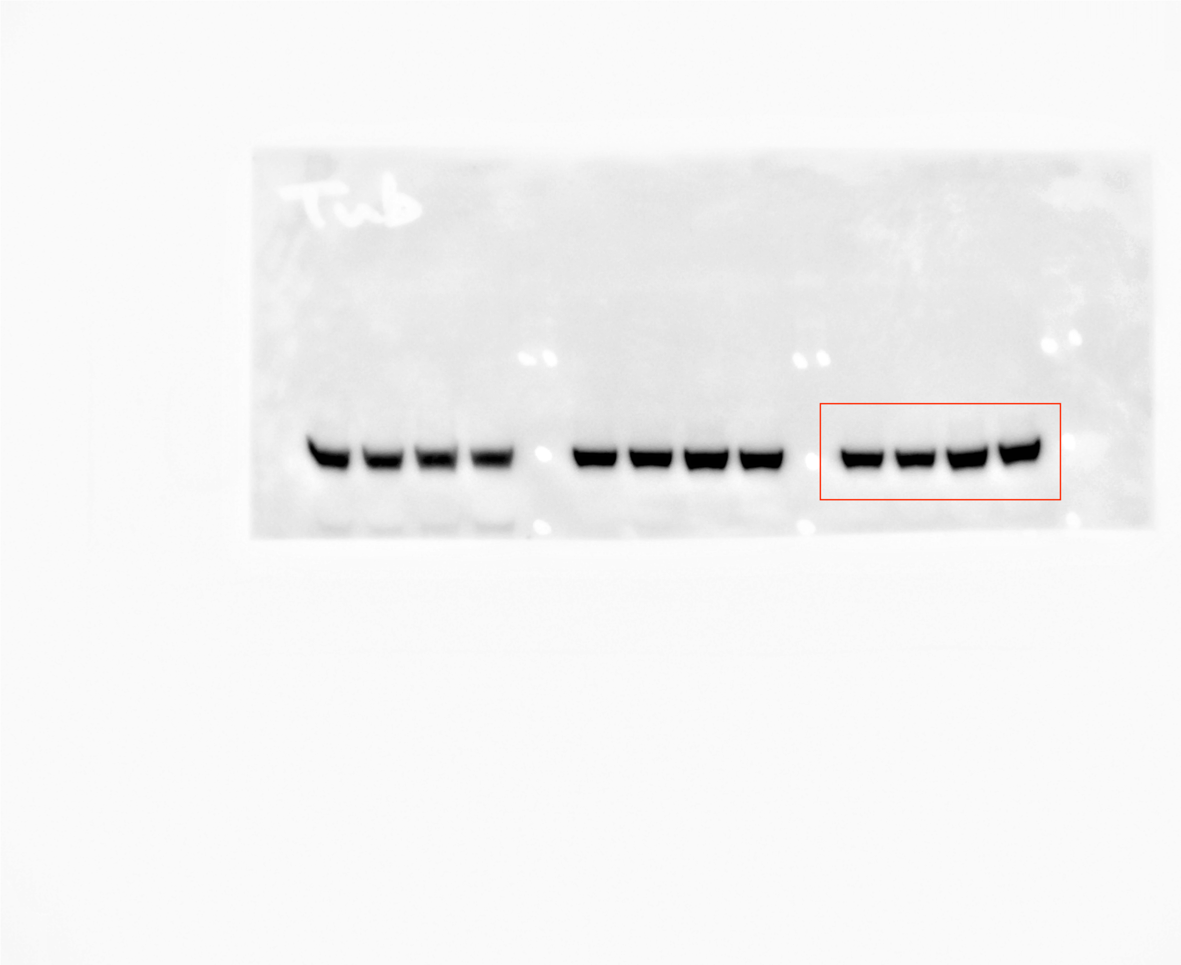

Supplemental Figure 8. Original blot for tubulin upon FcεRI stimulation.

The upper part of PVDF membrane used in Supplemental Figure 7 was stripped and subjected to western blot analysis against αtubulin. The image containing lanes 2-5 (red box) was cropped, horizontally flipped, and indicated as tubulin signal in Supplemental Figure 3a, bottom panel.
